# Detection of West Nile virus in wild birds in Tana River and Garissa Counties, Kenya

**DOI:** 10.1186/s12879-016-2019-8

**Published:** 2016-11-23

**Authors:** Doris Nyamwaya, Virginia Wang’ondu, Joshua Amimo, George Michuki, Moses Ogugo, Enoch Ontiri, Rosemary Sang, Johanna Lindahl, Delia Grace, Bernard Bett

**Affiliations:** 1 International Livestock Research Institute, P. O. Box 30709, 00100 Nairobi, Kenya; 2 Faculty of Veterinary Medicine, University of Nairobi, P. O. Box 29053, 00625 Nairobi, Kenya; 3 Department of Microbiology and Marine Botany, School of Biological Sciences, University of Nairobi, P. O. BOX 30197, 00100 Nairobi, Kenya; 4 U.S. Army Medical Research Unit (USAMRD-K), P.O. Box 606, 00621 Nairobi, Kenya

**Keywords:** Arbovirus, Flavivirus, Emerging infectious disease, West Nile fever, Zoonosis

## Abstract

**Background:**

West Nile fever virus is a zoonotic arboviral infection maintained in a sylvatic cycle involving mosquito vectors and birds. It is one the arboviruses whose geographical range is expanding because of climate and land use changes that enhance the densities of mosquitoes and promote mosquito-bird-human interactions. We carried out a survey to determine the reservoirs of WNV among wild birds in Tana River and Garissa counties, Kenya.

**Methods:**

Blood samples were obtained from 361 randomly trapped wild birds. Using real-time polymerase chain reaction (PCR), all samples were screened for WNV using gene specific primer sets amplifying a portion of the E region of the genome encoding the envelope protein.

**Results:**

Sixty five (65) out of 361 birds screened tested positive for WNV on real-time PCR assay. Sequencing of the selected positive samples reveals that the isolated WNV were most closely related to strains isolated from China (2011). A regression analysis indicated that sampling location influenced the occurrence of WNV while species, age, weight and sex of the birds did not have any effect.

**Conclusions:**

This study provides baseline information on the existing circulation of WNV in this region among wild bird reservoirs that could spill over to the human population and points to the need for implementation of surveillance programs to map the distribution of the virus among reservoirs. Awareness creation about West Nile fever in this region is important to improve its detection and management.

**Electronic supplementary material:**

The online version of this article (doi:10.1186/s12879-016-2019-8) contains supplementary material, which is available to authorized users.

## Background

West Nile virus (WNV) is a small single-stranded RNA virus classified under the genus flavivirus and grouped in the Japanese encephalitis virus sero-complex [1, 2]. It was first isolated from a febrile patient in Uganda [3] and is now one of the re-emerging zoonotic mosquito-borne pathogens whose occurrence has spread in a wide geographic range with major epidemics reported globally [4–6]. Similar to the other neurotropic flaviviruses, WNV is mainly transmitted by *Culex* mosquitoes with wild birds as the reservoirs and amplifying hosts [7, 8]. A higher competence among passerines has been demonstrated by their high susceptibility and ability to maintain high viremia for a prolonged duration [9]. While birds seems to be the most important reservoirs for WNV, antibodies againts the virus have also been detected in many mammals, some reptiles and amphibians [10], but the role of these species is not always understood. Humans and equines are incidental hosts as they produce insignificant viremia and do not contribute to WNV transmission. In most instances, the infection in human is mild and asymptomatic with less than 1% of patients developing meningoencephalitis, that often result in death [11].

Epidemics due to WNV have not been reported in Kenya. Studies have however demonstrated its presence in mosquitoes collected from the North Eastern Province [12, 13] and Turkwel gorge in the Rift Valley province where vertical transmission of the virus in *Culex univittatus* was observed [14]. *Culex quinquefasciatus, Cx. univittatus, and Cx. vansomereni* have been implicated as potential WNV vectors [15]. A sero-epidemiological survey that involved local communities in Tana River and Ijara Kenya, just before this study was initiated, indicated that people from irrigated areas were 1.27 times more likely to get exposed to WNV than those from pastoral areas [16]. A similar survey conducted in the local health centers among patients that sought treatment for febrile illnesses showed presence of WNV antibodies [16]. These studies indicated the presence and circulation of WNV in the region, and the potential importance of irrigation as a risk factor. This present study therefore sought to identify potential reservoirs (wild birds) of the virus to better understand its transmission cycle. This would provide insights for the development of surveillance, control and prevention strategies. The study was also designed to determine the lineage(s) of the WNV strains found in the area given that previous studies showed that both lineages 1 and 2 were present in the region.

## Methods

### Description of the study area

**Fig. 1:**
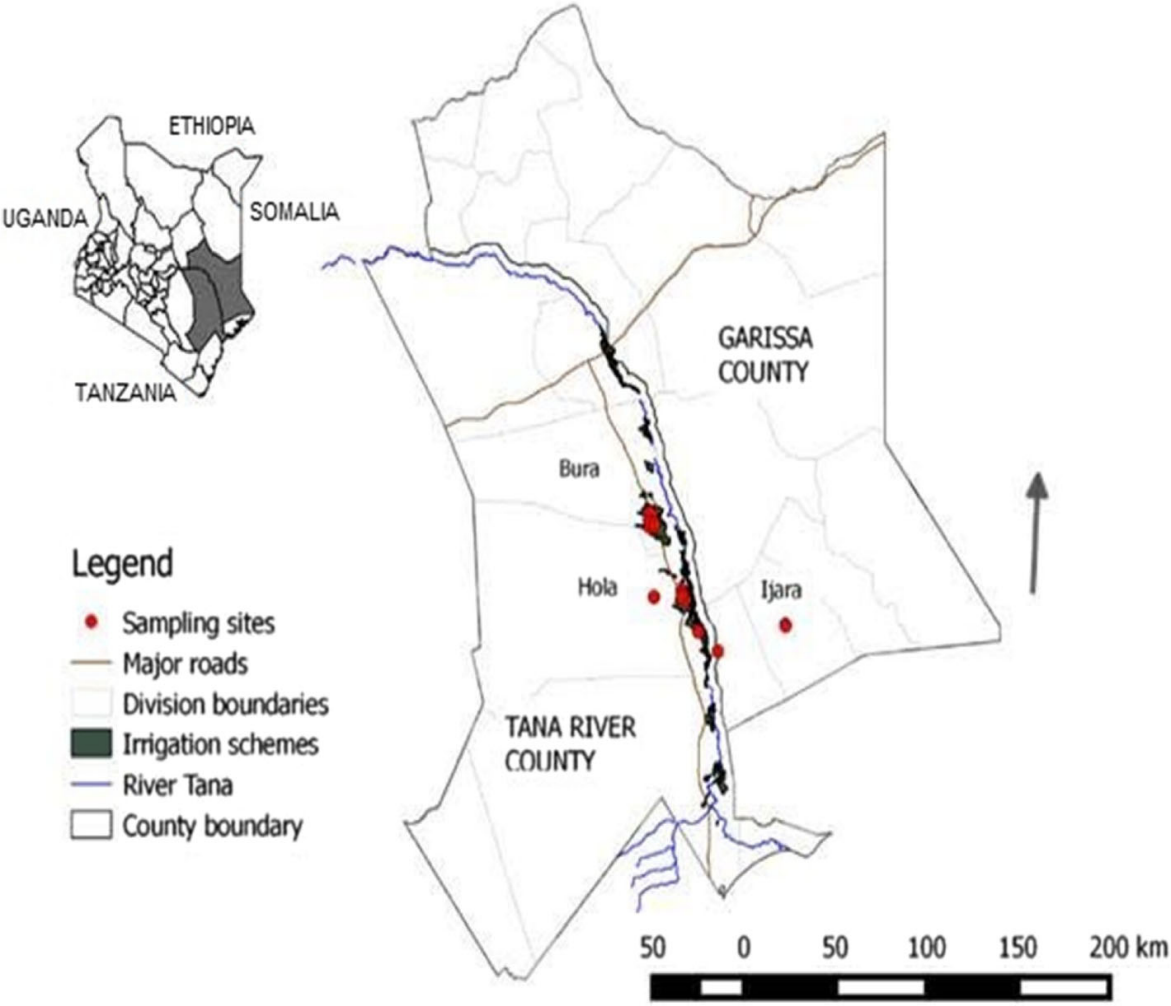
Bird trapping sites in Tana River County, Kenya

The study area traversed two neighboring counties – Tana River and Garissa -- in the eastern part of Kenya (Fig. 1). Tana River County is located between 38.45°–40.66° E and 3.04°–0.00° S while Garissa County is located between 38.67°–41.52°E and 2.04°–1.00° S. The area falls in an arid to semi-arid area with annual rainfall ranging between 400 and 750 mm and mean annual temperatures varying from 30 °c to 33 °c. It has a unique habitat diversity ranging from forests, woodlands, grasslands, riverine, mangroves, sand dunes and bushlands. The main economic activities in the area are farming and pastoralism. There are on-going large and small scale irrigation projects specifically in the sites falling in the Tana River County that produce maize, green grams and bananas among other food crops. Due to water pools in the irrigation schemes coupled with the hot and humid climatic conditions, there is a high mosquito density.

### Sampling

Birds were trapped in the dry and hot month of October 2014 and during the rainy season in December 2014. Cross sectional sampling was done for 14 days each occasion. Sites for bird trapping were selected randomly in Hola, Bura and Ijara. Mist nets (12 m length by 2 m height, 38 mm mesh and 6 panels) suspended 30 cm above the ground using bamboo poles 3 m long were used [17]. All Trapped birds were picked as soon as netting was observed to minimize traumatic stress on the trapped bird and the corresponding time was indicated. Extracted birds were placed in clean cotton bird bags in the shade and sampled according to standard ornithological bird handling and processing procedure [18]. Data recorded included species identification, estimated age, sex, and weight. The tail, tarsus, wing and head lengths were also recorded. Each bird was banded using a labeled ring from the National Museums of Kenya (NMK) for follow up purpose and to avoid re-sampling. Under aseptic conditions, blood was obtained from the brachial vein by a qualified and licensed ornithologist from NMK. In the month of October, blood samples were dispensed into sterile barcoded vials that were racked and stored in solid carbon dioxide (−78.5 °C). Due to the technical, logistical and cost challenges arising from the use of dry ice, a decision was made to switch to Whatman® FTA® card technology [19] for blood sample collection in December.

### Ribonucleic Acid (RNA) Extraction

Total RNA was extracted from blood using MagNA® Pure LC RNA Isolation Kit (Roche Diagnostics, Manneheim, Germany) according to the manufacturer’s instructions. Briefly, blood samples in vials were first thawed in ice and 200 μl transferred into a sample cartridge after a brief vortex. The FTA®card blood spots were punched out in small pieces, transferred into 1.5 ml sterile eppendorf tubes unto which 70 μl phosphate buffered saline (PBS) was added. After incubation for 5min at room temperature, 200μl of TriZol reagent was added and the contents vortexed for 3s then incubated at room temperature for 3 h before transferring to a sample cartridge for RNA extraction and elution. The quantity and purity of the eluted RNA after the automated extraction was determined using a NanoDrop® ND-1000 spectrophotometer (Thermo Scientific, Walton, Massachusetts, USA) before storage at - 80 °C until use.

### Reverse transcription

Complementary DNA was synthesized from the extracted RNA with SuperScript™ II (SS II) first strand synthesis system for RT-PCR (Invitrogen) following manufacturer’s instructions. For each reaction, 4 μl of sample total RNA was combined with 2 μl of random hexamers and heated at 65 °C for 5min then placed on ice immediately for 1min to anneal the primers to the 3′ terminal sequences of the RNA. SuperScript™ II reverse transcription mix was prepared according to the manufacturer’s instructions. Each reaction contained 4 μl 5X First strand buffer (250 mM Tris–HCl, pH 8.3 at room temperature, 375 Mm KCL, 15 mM MgCL_2_), 2 μl DTT (0.1 M), 2 μl Bovine Serum Albumin (10 mg/ml), 1 μl dNTPs(10 Mm), 0.2 μl RNase™ OUT(40U/μL) and 0.5 μl of SS II and then incubated for 2min at 25 °C. To each of the reaction wells containing heated RNA and primer mix, 9.7 μl of the prepared mix was added, gently pipetted up-down to mix contents and centrifuged at 12,000 rpm for 30s. The mix was annealed at 25 °C for 10min, extended at 42 °C for 15min and then the reverse transcription enzyme was inactivated by incubating for 15min at 70 °C then chilled at 4 °C.

### WNV screening

West Nile virus specific primers amplifying part of the highly conserved envelope-protein coding region of the WNV NY99 strain (reverse primer ‘1845’ of sequence 5′TTCCATCTTCACTCTACACT -3′ and forward primer ‘1401’ of sequence 5′ACCAACTACTGTGGAGTC -3′) [20] purchased from Bioneer® and diluted to a final working concentration of 20 pM in sterile water were used. Screening was done in a real-time polymerase chain reaction (PCR) against a standard curve generated by a synthetic WNV positive control (GenScript®). Into each well of a sterile labelled MicroAmp™fast 96 well reaction plate, 12.5 μL SyBr® Green master mix 0.5 μl of each primer (forward primer ‘1401’ and reverse primer ‘1845’), 10 μl of sterile PCR water (Bioline®) and 1.5 μl of synthesized cDNA were added. The plate was sealed using MicroAmp™ clear adhesive films (ABI) and loaded into 7900HT fast Real-time PCR system (Applied Biosystems). Thermal cycling conditions consisted of initial denaturation at 95 °C (10min), 35 cycles of denaturation at 95 °C (30s), annealing at 49 °C (45s) and extension at 72 °C (45s), a dissociation cycle 95 °C (15s), 60 °C (15s), 95 °C (15s) and a hold at 10 °C.

Sample amplification with a cycle threshold score of less than 40 was considered positive [21]. These products were analyzed by agarose gel (1.5% *w/v*) electrophoresis and visualized under UV illumination after GelRed™ (Biotium) staining. Samples with specific targeted 445 bp fragments were purified using a mini-elute purification kit (Qiagen) according to the manufacturer’ instructions and if the concentration was good quality with a concentration above 20 ng/μl submitted for Sanger sequencing using Big Dye Terminator Cycle chemistry and 3730 DNA Analyzer (Applied Biosystems, Foster, CA). The nucleotide sequences obtained from the selected strains were compared with the same segment of similar sequences of known strains available in GenBank using nBLAST (http://www.ncbi.nlm.nih.gov/BLAST/).

### Data analysis

In order to establish the relationship between the occurrences of WNV, the site of sampling and bird characteristics (age, sex and species), a regression analysis in a generalized linear model with a logit link implemented in R software was used. The Hosmer Lemeshow test was then used to test for the goodness of fit for this model. The confidence limit for the statistical tests was set at 95% (*P* < 0.05). The DNA sequences were aligned using a multiple sequence alignment program, Clustal Omega (http://www.ebi.ac.uk/Tools/msa/clustalo/) and the sequence identity calculated using the same program. Sequences with short read lengths below 200 bases were excluded from further analysis. The dendrogram was constructed using the neighbor-joining method supported with a bootstrap test of 1000 replicates in MEGA 6 software [22].

## Results

**Fig. 2:**
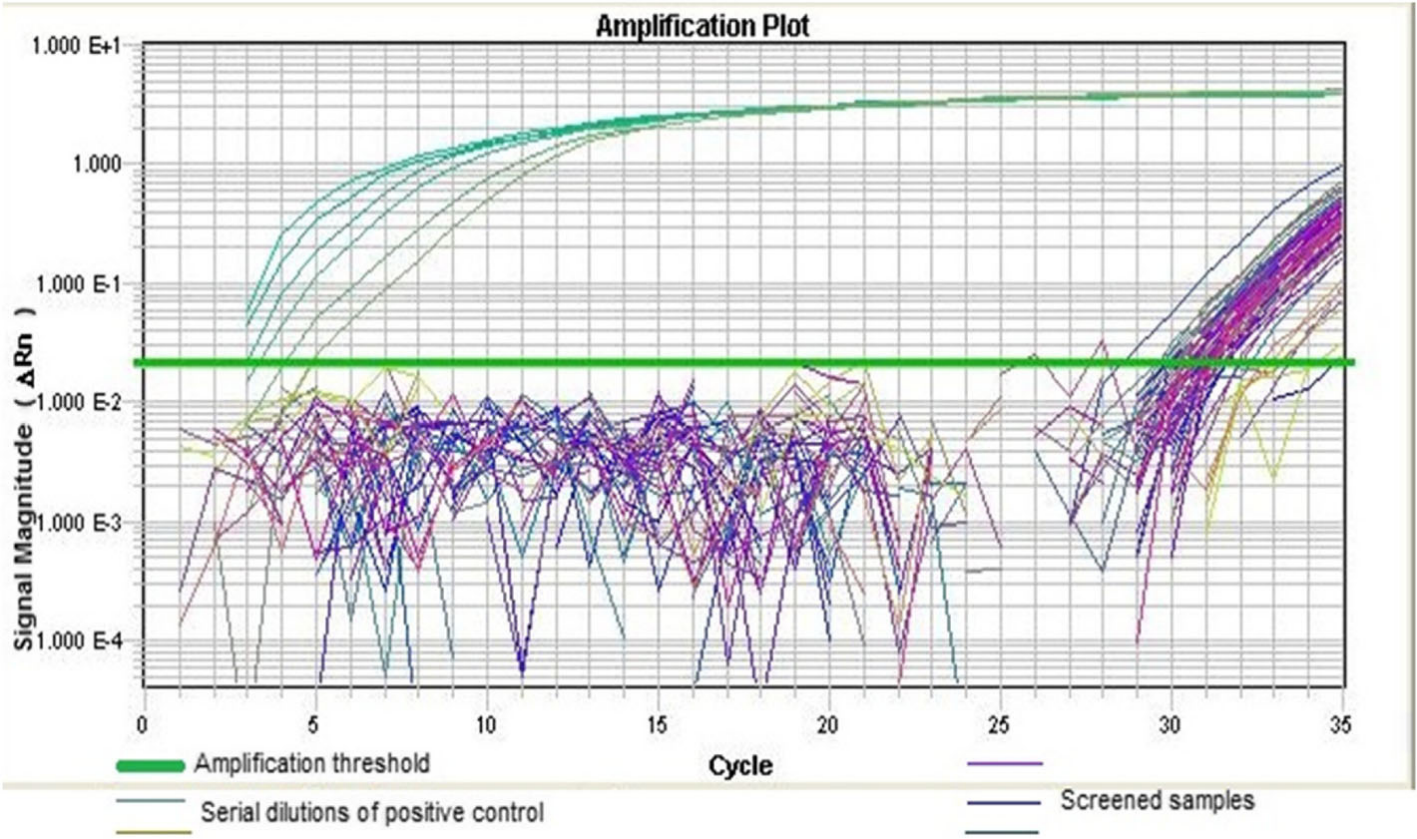
Fluorescence signal magnitude plot against PCR cycles generated from the real- time PCR: Serial dilutions of the positive control generated detectable amplification signals before the 5^th^ cycle. The screened samples that were positively amplified generated detectable amplification signals after the 25^th^ cycle. Positive samples had a CT value above 25

A total of 449 birds grouped into 61 species were trapped, of which 361 birds were sampled as the rest either escaped (0.7%) or were too weak to be bled (8.9%). The most frequently sampled species was the red-billed *Quelea (n = 81)*, lesser masked weaver birds (*n* = 57) and ring-necked doves (*n* = 34) (Additional file 1). All age groups and sex of birds were represented in the sample with most of the captured birds being adult males (*n* = 129). A total of 65 samples (18%) recorded a cycle threshold score of real time RT-PCR below 40 with a mean of 8.53^−11^(sd = 6.12^−11^) for detected WNV copies/ml and were considered positive (Fig. 2). In the month of December, only birds trapped from Bura tested positive, while there were infected birds from all the sampling sites in October (Table 1).

**Table 1:** Frequency distribution of bird species positive for WNV

Species	Frequency	WNV Positive Samples				
		BURA		HOLA	IJARA	Total
		October	December	October	October	
African golden weaver	8		2			2
African mourning dove	3		1		1	2
Barn swallow	5		2			2
Emerald spotted wood dove	9				2	2
Golden breasted starling	4				1	2
Golden pipit	8		2			2
Grey headed king fisher	3	1				2
House sparrow	22	4				2
Laughing dove	24		3	1		4
Lesser masked weaver	43		4	10		14
Namaqua dove	24		4	1		5
Nightjar	1				1	1
Nubian wood pecker	6				1	1
Red billed quelea	68	8	1	3		12
Ring necked dove	22		1		2	3
Ruppel’s long tailed starling	10				2	2
Violet backed sunbird	1		1			1
White browed sparrow weaver	2	1				1
White headed buffalo weaver	15	1			3	4
White throated bee eater	2		1			1
Total		15	22	15	13	65

A regression analysis using a generalized linear model which included bird’s age, sex, region of capture and species as independent factors showed that only the region of capture had a significant effect (*p* = 0.01) on WNV occurrence with birds from Hola and Ijara being less likely to harbor the virus compared to birds from Bura where there are more extensive irrigation schemes.

After PCR product purification, 35 samples were good quality and were sequenced. Except for 3 sequences with read lengths between 200 and 450 bases, the rest had short read lengths and were excluded from further analysis. A similarity search using nBLAST tool in the NCBI database revealed a high similarity between Tana River WNV strains and WNV strains detected in China (2011) (GeneBank accession numbers: *XJ11141, XJ11129* and *XJ11148*). A sequence identity comparison table generated by incorporating retrieved sequences of WNV isolates from the Genebank (NCBI) and sequences from this study (accession numbers; KX189175, KX189174 and KX189176) showed differential similarity of Tana River strains to other WNV isolated strains. Identity to published strains grouped under Lineage I were all above 95% except for Kenya tick strain, while sequence similarity between our samples and lineage II -IV was below 70% (Table 2).

**Table 2:** Percentage nucleotide identity between WNV isolates available in Genebank and Tana River WNV strains

WNV STRAINS	Lineage	KX189174_R.b.quelea_2D_2016_Bura_Kenya	KX189175_Dove_4A_2016_Bura_Kenya	KX189176_Dove_4B_2016_Bura_Kenya
DQ118127_goose-Hungary/03	I	96.9	95.2	96.9
KJ786934_NY2001-6263_Homosapiens	I	97.6	95.9	96.9
KT163243_68856-ICDC-4_2015_India	I	95.9	94.1	95.2
JX442279_XJ11129_C.pipiens_2011_China	I	100.0	97.9	99.3
JX442281_XJ11141_C.pipiens_2011_China	I	100.0	97.9	99.3
DQ374650_Ast02-3-717_P.carbo_2006_Russia	I	99.7	97.9	99.0
DQ411031_Ast01-187_C.corone_2006_Russia	I	99.7	97.9	99.0
DQ411030_Ast01-182_H.Marginatum_2006_Russia	I	99.7	97.9	99.0
KJ501417_WNV-1/US/BID-V6684/2006	I	98.3	96.2	97.6
EF631149_CpWw21_CpWw21_Cx.pipiens_2006_USA	I	98.3	96.2	97.6
HM538818_4893_2007_USA_linaege 2	I	98.3	96.2	97.6
DQ786573_France407/04_C.magpie_2004_France	I	96.2	94.5	95.5
AY712947_Bird1461_2004_Tx_USA	I	97.9	96.2	97.2
AY052409_Goose_ISR98-GooKha_1998_Israel	I	97.2	95.5	96.6
KC243146_tick_ATH002316_2006_Kenya	I	92.8	91.0	92.1
**KX189174_R.b.quelea_2D_2016_Bura_Kenya**	I	**100.0**	**97.9**	**99.3**
**KX189175_Dove_4A_2016_Bura_Kenya**	I	**97.9**	**100.0**	**97.2**
**KX189176_Dove_4B_2016_Bura_Kenya**	I	**99.3**	**97.2**	**100.0**
DQ318019_WNV_ArD76104_2006_Senegal	II	75.5	73.8	75.5
HM147822_WNV_1958_SAfrica_lineage2	II	75.2	73.8	75.2
EF429199_human_SA381/00_2000_S.Africa	II	76.9	75.2	76.9
GQ903680_Q3574-5_1968_Cyprus	II	75.3	73.9	75.3
HM147824_1958_DRCongo	II	76.6	74.8	76.6
AY688948_Sarafend_2005_Israel	II	76.2	74.8	76.2
AY765264_Rabensburg_C.pipiens_Czech_lineage 3	III	77.2	75.2	77.2
AY277251_LEIV-Krnd88-190_D.marginatus_1998_Russia_Lineage 4	IV	71.7	70.0	71.0
JN638336_Denguevirus1_KD86-035_1986_outgroup		53.0	51.6	52.3

In bold are sequences obtained from this particular study. Other sequences used in generating the identity matrix were obtained from the genebank

**Fig. 3:**
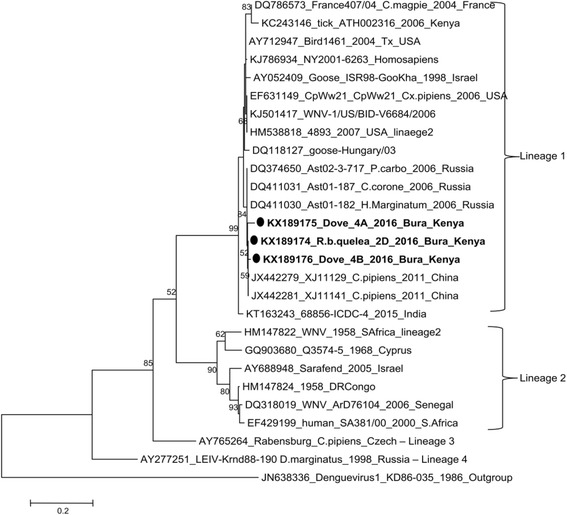
Molecular phylogenetic analysis by Maximum Likelihood method between Kenyan WNV bird strains (bold) and known WNV strains in the GenBank. The evolutionary history was inferred by using the Maximum Likelihood method based on the Tajima-Nei model [35]. The tree with the highest log likelihood (−1866.0300) is shown. The percentage of trees in which the associated taxa clustered together is shown next to the branches. Initial tree(s) for the heuristic search were obtained automatically by applying Neighbor-Join and BioNJ algorithms to a matrix of pairwise distances estimated using the Maximum Composite Likelihood (MCL) approach, and then selecting the topology with superior log likelihood value. Evolutionary analyses were conducted in MEGA6 [22]

**Fig. 4:**
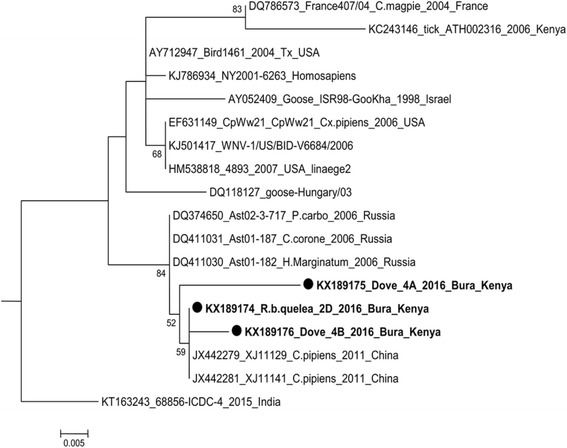
Phylogenetic tree of WNV lineage 1 strains : The lineage includes different evolutionary clades. Tana River strains (accession numbers: KX189175, KX189174 and KX189176) clustered together indicating that they are closely related

Phylogenetic analysis by maximum likelihood method conducted in MEGA6 [22] incorporating other published WNV strains available in GenBank with strains in this study, further confirmed the close evolutionary relationship to WNV strains described as Lineage 1 [23] (Figs. 3 and 4).

## Discussion

This study shows the circulation of WNV among different Kenyan bird species; this information is needed to further understand the risks and epidemiology of the virus in the region. West Nile virus was first detected in East Africa and although the virus has been known for long, there is no established vaccination or curative regimes, it remains a pathogen of public health concern in Africa. After its first isolation in Uganda (1937), a sero-survey (1939–1940) showed a widespread human sero-positivity in Uganda, Kenya, Democratic Republic of Congo and Sudan [24]. In 1950, WNV was isolated for the second time in Egypt during a sero-survey that demonstrated presence of WNV neutralizing antibodies in 70% of participants [25] indicating a widespread transmission in the local population. West Nile virus is endemic in Kenya and has been isolated from mosquitoes [12, 13] and ticks *(Rhipicephalus pulchellus)* [26] in northeastern Kenya. This study has provided information on the avian amplification hosts that may be significant in maintaining the pathogen in nature.

Tana River County is a rich avifaunal destination supporting both local and migratory birds as revealed by the high diversity of sampled birds in this study. Bird species as a factor did not influence the occurrence of WNV in Tana River, concurring with studies which showed that transmission of WNV in the old world was dependent on presence of a carrier avian host, ornithophilic vectors and inter-species infection of numerous bird amplifying hosts in a given locality [27] independent of the bird species. Despite the 1999 WNV outbreak in New York being characterized by massive deaths of American crows (*Corvus brachyrhynchos*) [28], passerine bird species have been implicated as the most competent WNV hosts in Africa [29, 30] as they develop high viremia levels with low mortality [9] that allow for transmission to competent mosquito vectors. This is confirmed in this study as more passerine species screened were positive for WNV. Presence of WNV in doves was observed, however, they have been described in previous studies in Egypt and South Africa to be only weakly competent amplifiers of WNV [29, 30].

Despite improving food security in the semi-arid county, irrigation activity has led to increased mosquito densities and occurrence of zoonotic pathogens including WNV [16]. In this study, there were more WNV positive bird blood samples from Bura, a region that has a larger irrigated area in comparison to other sampled regions and is likely to attract more birds due to availability of a higher food base and high mosquito density. This was the same trend observed with sero-positivity to WNV during surveillance studies of human blood samples in this locality with patients from irrigated sites exhibiting high prevalence rates [16].

The geographic and genetic diversity of WNV has been previously described with five distinct lineages proposed from phylogenetic analyses of various isolates [23, 31, 32]. Most human outbreaks have been associated with lineages 1, 2 and 5 [23, 33]. Lineage 1 isolates have a wide geographic distribution globally and have evolved into clades 1a and 1b that have been divided further into subclades (Fig. 4). Despite WNV strains from Tana River County exhibiting high similarity to lineage 1 WNV strains (ranging from 80 to 96%), they tended to be more similar to each other when compared to those isolated from different geographical localities (Fig. 4). This would be an indication that a particular strain of WNV was introduced to this locality by an infected migratory bird [6] and its existence has been maintained through amplification in local resident bird species and transmission through diverse mosquito species. Phylogenetic analysis of selected sequences revealed that WNV strain from the study region is genetically most closely related to WNV strains *XJ11141 and XJ11129* isolated in 2011 from mosquitoes in Xinjiang Uyghur, western China [34]. The E gene from the Xinjiang strains showed a high degree of genetic identity of lineage 1 with other highly pathogenic WNV strains, such as Ast01-182 from Russia [34]. This would be a confirmation to the rapid transmission and distribution of the virus globally.

## Conclusion

This study confirms circulation of WNV among wild birds in Tana River, Kenya. Availability of good breeding grounds for mosquito vectors and adequate food base for birds in this county are among factors that would enhance propagation of this pathogen. Since the virus has been reported to rapidly expand its geographical range and epidemic development, there is need to promote awareness in the public health departments and among local residents in order to prevent fatal outbreaks. There is inadequate data on surveillance for active WNV infection in birds and this study therefore provides a baseline for further research to enable adequate comprehension of the ecology of this pathogen in Kenya and assist a potential statement of public health measures to avoid incidents of morbidity and mortality due to sudden disease outbreaks. Although the majority of WNV infections in humans produce mild infections, no studies have been done to demonstrate the virulence of the virus when it infects an immune-compromised individual or when it infect patients with concurrent infections. Tana River provides a roosting (stopover) habitat for migratory birds moving from northern Europe and beyond to the south of the African continent, explaining the flow of virus genes from the north to Tana River. In addition, domestic and wild animals converge here to water which would provide opportunity for further evolutionary changes and emergence of more lineages.

These possibilities suggest an urgent need to conduct more intensive surveillance to determine the intensity of WNV transmission in the area.

## Additional file

Additional file 1: Description: List of Bird Species captured in Tana River County, Kenya. Title: Frequency table of sampled bird species. (DOCX 18 kb)

## Abbreviations

cDNA: Complementary deoxyribonucleic acid
dNTPs: Deoxynucleotides
MEGA: Molecular Evolutionary Genetics Analysis
NCBI: National Center for Biotechnology Information
NMK: National Museums of Kenya
PCR: Reverse transcription polymerase chain reaction
RNA: Ribonucleic acid
UV: Ultraviolet
WNV: West Nile virus
