# Supplementary material for: Detection of West Nile virus in wild birds in Tana River and Garissa Counties, Kenya
Source: BMC Infect Dis. 2016 Nov 23;16:696. doi: 10.1186/s12879-016-2019-8 (PMC5121970; doi:10.1186/s12879-016-2019-8)
Supplement: Additional file 1: — Description: List of Bird Species captured in Tana River County, Kenya. Title: Frequency table of sampled bird species. (DOCX 18 kb) [file 12879_2016_2019_MOESM1_ESM.docx]

List of Bird Species captured in Tana River and Garissa Counties

|  | **Species** | **Sampling Sites** | | | |
| --- | --- | --- | --- | --- | --- |
|  |  | **Bura** | **Hola** | **Ijara** | **Total** |
| 1 | 3 streaked tchagra |  | 1 |  | 1 |
| 2 | african golden weaver | 7 | 1 |  | 8 |
| 3 | african mourning dove | 1 |  | 2 | 3 |
| 4 | african palm swift | 1 |  |  | 1 |
| 5 | barn swallow | 5 |  |  | 5 |
| 6 | black headed plover | 2 |  |  | 2 |
| 7 | blue naped mousebird |  | 1 |  | 1 |
| 8 | brown throated barbet |  | 3 |  | 3 |
| 9 | chestnut weaver | 2 |  |  | 2 |
| 10 | common bulbul |  | 3 |  | 3 |
| 11 | d'arnaud's barbet |  | 3 |  | 3 |
| 12 | diederick cuckoo | 1 |  |  | 1 |
| 13 | eastern violet backed sunbird |  | 3 |  | 3 |
| 14 | emerald spotted wood dove |  | 5 | 5 | 10 |
| 15 | fire fronted bishop | 26 | 5 |  | 31 |
| 16 | fischer's starling | 3 | 5 | 2 | 10 |
| 17 | golden breasted starling | 3 |  | 1 | 4 |
| 18 | golden pipit | 8 |  |  | 8 |
| 19 | green winged pytilia |  | 4 |  | 4 |
| 20 | grey headed king fisher | 4 |  |  | 4 |
| 21 | grey headed sparrow |  |  | 2 | 2 |
| 22 | harlequin quail | 3 |  |  | 3 |
| 23 | house sparrow | 15 | 12 |  | 27 |
| 24 | laughing dove | 21 | 3 |  | 24 |
| 25 | lesser honey guide | 2 |  |  | 2 |
| 26 | lesser masked weaver | 20 | 37 |  | 57 |
| 27 | magpie starling |  |  | 1 | 1 |
| 28 | malachite king fisher | 2 |  |  | 2 |
| 29 | namaqua dove | 19 | 5 |  | 24 |
| 30 | nightjar |  |  | 1 | 1 |
| 31 | northern brown bul |  | 4 |  | 4 |
| 32 | northern crombec |  | 3 |  | 3 |
| 33 | northern wheat ear |  | 1 |  | 1 |
| 34 | northern white crowned shrike | 1 |  |  | 1 |
| 35 | nubian wood pecker | 1 | 2 | 3 | 6 |
| 36 | olivaceous warbler |  | 1 |  | 1 |
| 37 | pale wren warbler |  | 4 |  | 4 |
| 38 | pink breasted lark |  | 1 |  | 1 |
| 39 | red and yellow barbet | 1 |  |  | 1 |
| 40 | red billed buffalo weaver |  |  | 3 | 3 |
| 41 | red billed quilea | 44 | 36 | 1 | 81 |
| 42 | red fronted warbler | 1 | 1 |  | 2 |
| 43 | red tailed shrike |  | 2 |  | 2 |
| 44 | ring necked dove | 1 | 4 | 17 | 22 |
| 45 | rosy patched bush shrike |  | 2 |  | 2 |
| 46 | rufous chatterer |  | 2 |  | 2 |
| 47 | ruppel's long tailed starling |  |  | 10 | 10 |
| 48 | somali bee eater |  | 1 |  | 1 |
| 49 | speckled mousebird |  | 3 |  | 3 |
| 50 | spur winged plover | 1 |  |  | 1 |
| 51 | superb starling |  | 1 | 3 | 4 |
| 52 | upcher's warbler |  | 1 |  | 1 |
| 53 | violet backed sunbird | 1 |  |  | 1 |
| 54 | violet breasted sunbird | 2 | 7 |  | 9 |
| 55 | von-der-decken's |  |  | 1 | 1 |
| 56 | white browed scrub robin |  | 7 |  | 7 |
| 57 | white browed sparrow weaver | 4 |  |  | 4 |
| 58 | white eared bulbul |  | 1 |  | 1 |
| 59 | white headed buffalo weaver | 2 | 8 | 7 | 17 |
| 60 | white throated bee eater | 1 |  |  | 1 |
| 61 | willow warbler | 2 |  |  | 2 |
|  | **Total** | **207** | **183** | **59** | **449** |
